# Single-Molecule Long-Read Sequencing of Purslane (*Portulaca oleracea*) and Differential Gene Expression Related with Biosynthesis of Unsaturated Fatty Acids

**DOI:** 10.3390/plants10040655

**Published:** 2021-03-30

**Authors:** Hongmei Du, Shah Zaman, Shuiqingqing Hu, Shengquan Che

**Affiliations:** 1 School of Design, Shanghai Jiao Tong University, Shanghai 200240, China; hmdu@sjtu.edu.cn (H.D.); hushuiqingqing@sjtu.edu.cn (S.H.); 2 School of Agricultural and Biology, Shanghai Jiao Tong University, Shanghai 200240, China; Shah_Zaman@sjtu.edu.cn

**Keywords:** purslane, RNA-seq, gene, unsaturated fatty acid

## Abstract

This study aimed to obtain the full-length transcriptome of purslane (*Portulaca oleracea*); assorted plant samples were used for single-molecule real-time (SMRT) sequencing. Based on SMRT, functional annotation of transcripts, transcript factors (TFs) analysis, simple sequence repeat analysis and long non-coding RNAs (LncRNAs) prediction were accomplished. Total 15.33-GB reads were produced; with 9,350,222 subreads and the average length of subreads, 1640 bp was counted. With 99.99% accuracy, after clustering, 132,536 transcripts and 78,559 genes were detected. All unique SMART transcripts were annotated in seven functional databases. 4180 TFs (including transcript regulators) and 7289 LncRNAs were predicted. The results of RNA-seq were confirmed with qRT–PCR analysis. Illumina sequencing of leaves and roots of two purslane genotypes was carried out. Amounts of differential expression genes and related KEGG pathways were found. The expression profiles of related genes in the biosynthesis of unsaturated fatty acids pathway in leaves and roots of two genotypes of purslane were analyzed. Differential expression of genes in this pathway built the foundation of ω-3 fatty acid accumulation in different organs and genotypes of purslane. The aforementioned results provide sequence information and may be a valuable resource for whole-genome sequencing of purslane in the future.

## 1. Introduction

Purslane (*Portulaca oleracea*) first came from India and Iran and has spread around the world. It is a warm-climate, juicy annual plant spread around the world, belonging to the Portulacaceae family. Purslane is one of the most abundant terrestrial vegetables in spite of its genetic assortment [1,2,3] and generally recognized as purslane (the USA and Australia), Ma-Chi-Xian (China) [4] and kurfa in (Pakistan) [5,6]. It has been used as an important traditional medicinal plant and itemized by the World Health Organization as “Global Panacea” [7].

Apart from medicinal and nutritive value, purslane is a high-quality halophyte [8]. Some results have been reported about its salt tolerance mechanism [9,10,11,12]. Since, the development of high-throughput sequencing technology, transcripts sequencing has become an important means to study gene expression regulation, after the whole-genome sequencing of human were completed in 2004 [13]. However, due to the read length limitation of the second-generation sequencing in different organisms [14,15,16,17,18], the full-length transcript obtained by splicing is not complete. The third-generation sequencing technology signified by Pacific Biosciences (PacBio, Menlo Park, CA, USA) effectively overcome this problem [19,20]. Single-molecule real-time (SMRT) sequencing can directly obtain full-length splice isoforms without the need for assembly [21,22], improve the draft genome annotation in species with reference genome and facilitate comparative transcriptome studies and gene functional annotation [22]. Up to date, this technology has been successfully utilized in some species, such as perennial ryegrass (*Lolium perenne*) [22], *Rhododendron lapponicum* [23], strawberry (*Fragaria* × *ananassa*) [24], *Gnetum luofuense* [25], and maize (*Zea mays*) [21]. However, up to date, no researcher studied the full-length transcript of purslane using single-molecule long-read sequencing. In this study, SMRT sequencing was achieved in purslane. After detecting transcripts, we completed functional annotations of transcripts, transcript factors (TFs) and simple sequence repeat (SSR) analysis, long non-coding RNAs (LncRNA) prediction. This study established a high-quality reference transcriptome for purslane, which provides valuable resources for further investigation of related molecular mechanisms, especially biosynthesis of unsaturated fatty acids pathway in purslane.

## 2. Results

### 2.1. Single-Molecule Real-Time Sequencing of Purslane

**Figure 1:**
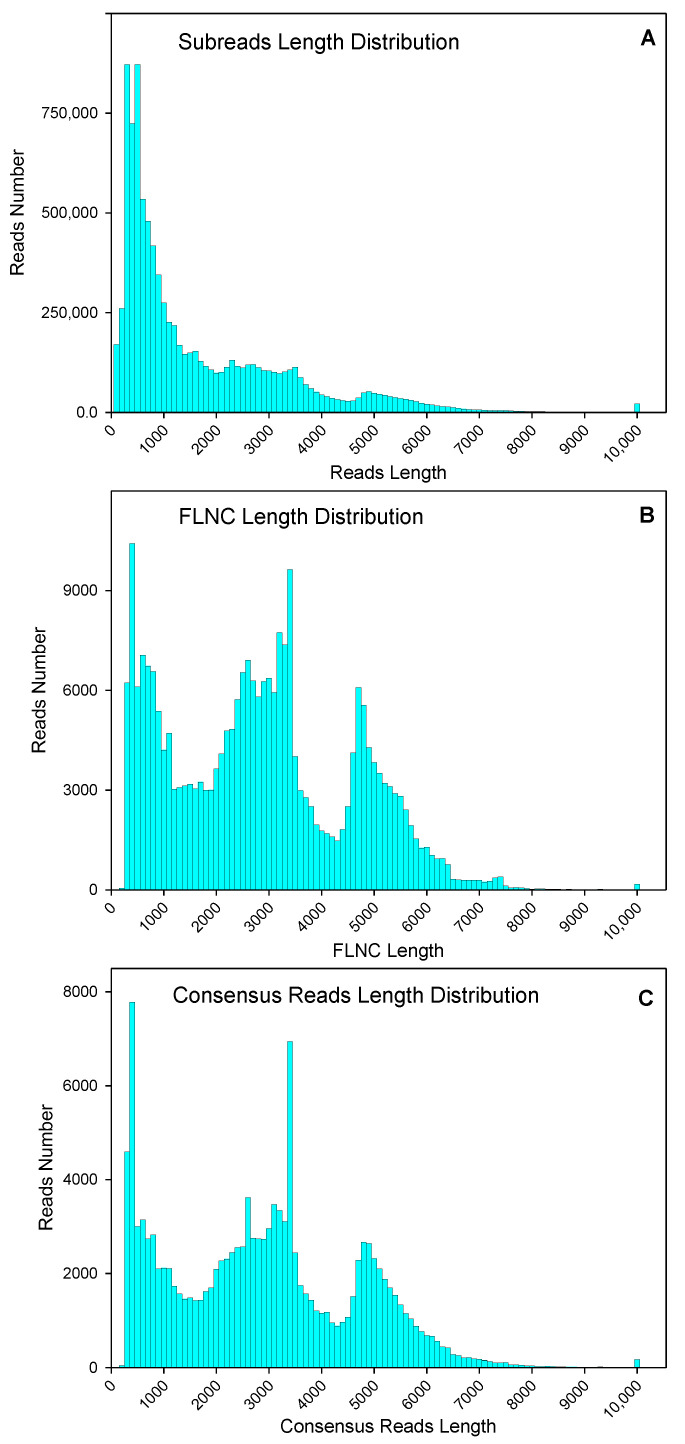
Numbers and length distributions of 9,350,222 subreads (**A**), 254,692 full-length non-chimeric (FLNC) sequences (**B**), and 132,536 consensus reads (**C**) with PacBio single-molecule real-time sequencing method of purslane (*Portulaca oleracea*).

Leaves and roots from “Pakistan local” (“PL”-North American origin) and a wild variety “Liaoning, China local” (“LCL”) were used for RNA extraction and cDNA library construction. After removing adaptor sequences, low-quality sequences, and short sequences less than 50 bp, a total of 9,350,222 subread (15.33 Gb) were obtained, through normal subread length of 1640 bp, and N50, of 3093 bp (Figure 1A). After self-correction of subread sequences (with min passes = 2, min predicted accuracy = 0.8), a total of 375,102 circular consensus sequence (CCS) were obtained. After sequencing, 259,265 full-length and 254,692 full-length non-chimeric (FLNC) picks were identified. The average FLNC read length was 2808 bp (Figure 1B). The FLNC sequences of the same transcript were clustered using the iterative isoform-clustering (ICE) algorithm, and 132,536 consensus reads were obtained after clustering (Figure 1C).

**Figure 2:**
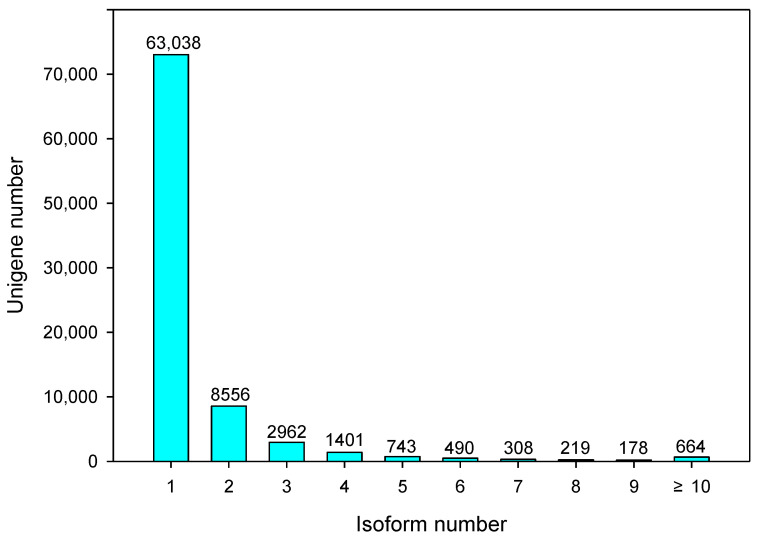
Number of isoforms in purslane (*Portulaca oleracea*).

The number of genes with no isoform remained 53,977, and 78,559 transcriptions were found between 1 and 10 isoforms (Figure 2). The transcript length extended from 170 bp to 14,287 bp, with an average length of 3061 bp.

### 2.2. Functional Annotation of Full-Length Transcripts

**Figure 3:**
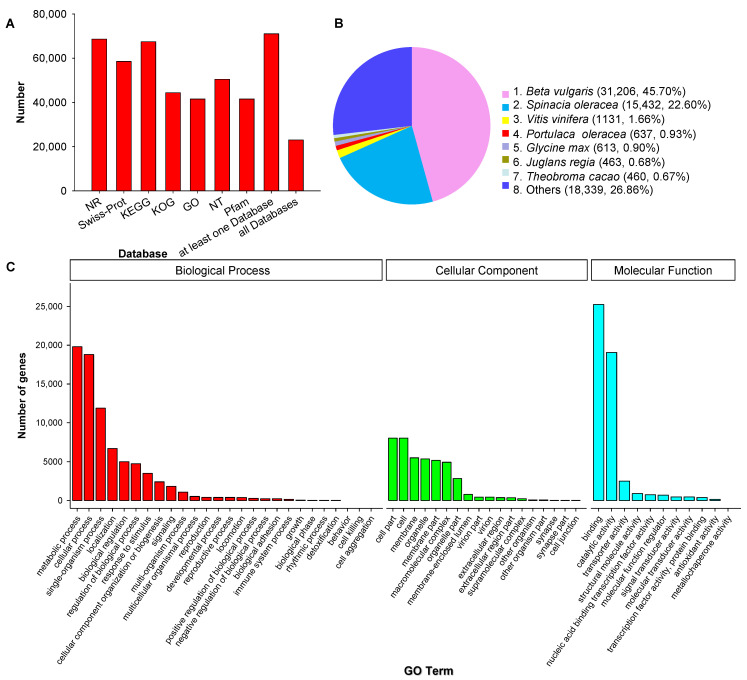
Function annotation of corrected isoforms in seven databases (**A**) (NR, NCBI Nonredundant Protein Database; KEGG, Kyoto Encyclopedia of Genes and Genomes; KOG, cluster of eukaryotic ortholog groups of proteins; GO, Gene Ontology; NT, NCBI nucleotide sequences; Pfam, Protein family), homologous species distribution diagram of transcripts in NCBI nonredundant protein sequences (NR) (**B**), Gene Ontology (GO) classification of unique transcripts (**C**) of purslane (*Portulaca oleracea*).

All 78,559 unique SMRT transcripts were functionally annotated by searching seven data storage, including Gene Ontology (GO), eukaryotic ortholog groups (KOG), protein family (Pfam), NCBI nucleotide sequences (NT), NCBI nonredundant protein sequences (NR), Swiss-Prot, Kyoto Encyclopedia of Genes and Genomes (KEGG). 90.39% transcript, a total of 71,008, was annotated at one database at least, and 23,013 transcripts were annotated at all seven databases (Figure 3A). We also identified matches to our unique transcripts in clusters of orthologous groups of proteins (COG) (44,376, 56.49%), Pfam database (41,535, 52.87%) and Swiss-Port (58,535, 74.51%). The functional annotations of all 78,559 unique transcripts were listed in Supplementary Table S1.

We compared the transcript sequences to NR by homologous species analysis. 68,630 genes were annotated, among them, *Beta valgaris* (31,206; 45.70%), *Spinacia oleracea* (15,432; 22.60%), *Vitis vinifera* (1131; 1.66%), and *P. oleracea* (637; 0.93%) were the top four species of transcripts distributed (Figure 3B). GO analysis demonstrated that 41,535 unique genes were enriched significantly in three major categories: molecular function (MF), cellular component (CC), biological process (BP). In these three categories, the most abundant GO terms were cellular process and metabolic process in BP, catalytic activity and binding in MF, cell and cell part in CC (Figure 3C).

**Figure 4:**
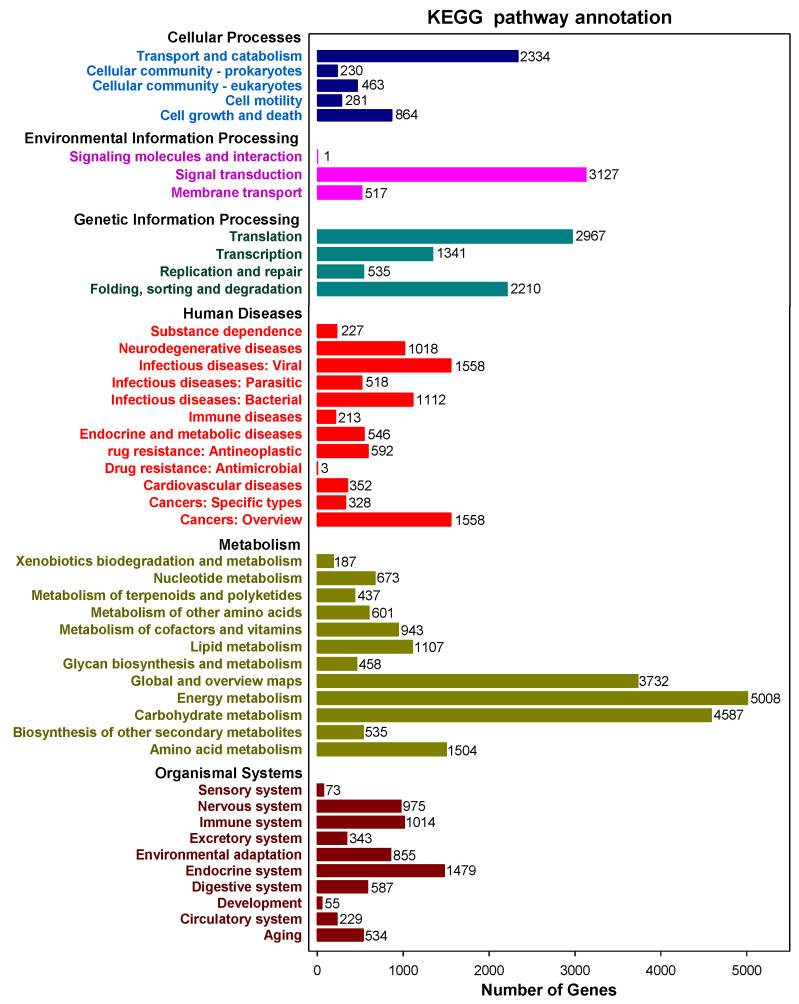
Kyoto Encyclopedia of Genes and Genomes (KEGG) annotated pathways and numbers of the gene in purslane (*Portulaca oleracea*).

A total of 67,426 sequences were interpreted by the KEGG data storage and plotted to 366 operative catalogs in purslane. “Metabolism” was the largest transcript category. The first three transcripts-related pathways were carbon metabolism (3007, 4.46%), carbon fixation in photosynthetic organisms (2304, 3.42%) and pyruvate metabolism (2153, 3.19%) (Figure 4 and Supplementary Table S1). A large number of genes, especially interrelated in salt-tolerance and the fatty acid component of purslane, were annotated, such as oxidative phosphorylation (l073), plant hormone signal transduction (506), fatty acid biosynthesis (246), biosynthesis of unsaturated fatty acids (94) and α-linolenic acid metabolism (199) (Supplementary Table S1).

### 2.3. Results of Transcript Factors, Long Non-Coding RNAs and Simple Sequence Repeat

**Figure 5:**
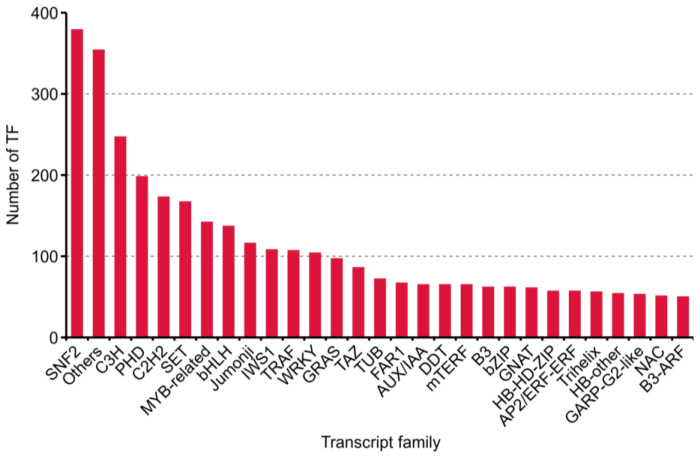
Numbers and families of the top 30 transcript factors (TFs) predicted by iTAK software in purslane (*Portulaca oleracea*).

During plant growth and development, TFs and transcription regulation (TRs) acting as dominant characters. Four thousand one hundred eighty transcripts, including 2211 putative TF and 1969 TR from 86 families, were predicted with iTAK software [26] (Supplementary Table S2). The top 30 families annotated are shown in Figure 5.

**Figure 6:**
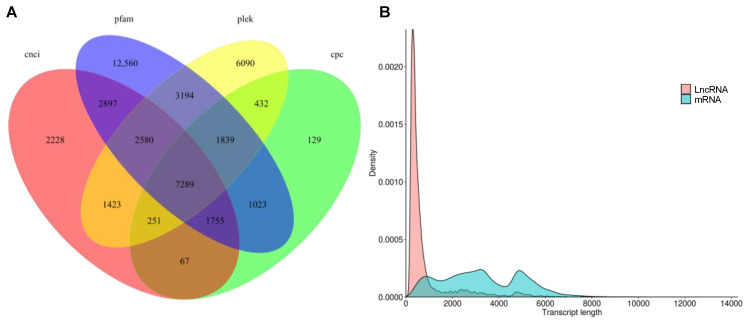
Identification of long non-coding RNAs (LncRNAs). (**A**). Venn diagram of LncRNAs predicted by PLEK, coding-non-coding index (CNCI), coding potential calculator (CPC) and protein family (Pfam) methods. (**B**). Density and length distributions of LncRNAs and mRNAs in purslane (*Portulaca oleracea*).

LncRNA were predicted by PLEK, Pfam, coding potential calculator (CPC) and coding-non-coding index (CNCI) (Figure 6A). Overall, 7289 LncRNA were predicted with a mean length of 848.84 bp with most LncRNAs length ranging from 300 bp to 1000 bp (Figure 6B and Supplementary Table S3).

SSR is also known as short tandem repeats or microsatellite markers. A total of 58,622 sequences were subjected to SSR analysis. Most of the SSRs identified were one-nucleotide repeats (50.81%), followed by compound nucleotide repeats (17.11%), two-nucleotide repeats (15.20%), three-nucleotide repeats (14.76%), four-nucleotide repeats (1.16%), six-nucleotide repeats (0.73%), and five-nucleotide repeats (0.40%) (Table S4).

### 2.4. qRT–PCR Validation of Selected Genes

**Figure 7:**
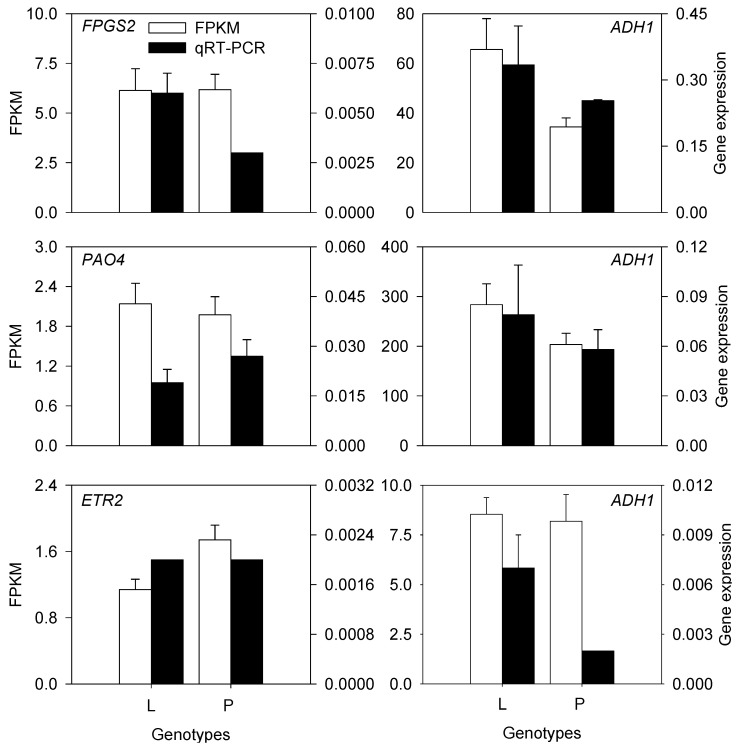
Validation of the mRNA expression levels of a gene by RNA-seq and qRT–PCR analyses.

To confirm the results of RNA-seq, we executed qRT-PCR with 6 genes in 4 KEGG pathways, including 1 *FPGS2* in folate biosynthesis, 1 *PAO4* in arginine and proline metabolism, 1 *ETR2* in plant hormone signal transduction and 3 *ADH1* in α-linolenic acid metabolism. Gene expression results with qRT-PCR verification showed a very similar tendency with Fragments per kilobase of transcript per million mapped reads (FPKM) values from sequencing at the same conditions (Figure 7), which indicated that the RNA-seq data were reliable.

### 2.5. Illumina Sequencing Results and Differential Gene Expression in Leaves and Roots of Two Purslane Genotypes

Illumina sequencing was performed on leaves or roots of two purslane genotypes, separately, with three independent biological replicates. Clean reads of leave and root samples of two genotypes are shown in Table 1. These sequences were mapped to reference sequence (REF) of purslane for annotation of all unigenes. The mapping rate was 57.59%–76.84% for all samples in two purslane genotypes (Table 1). Compared with “LCL”, 9099 and 7408 differentially expressed genes (DEGs) were found upregulated, 9550 and 6802 DEGs downregulated in leaves and roots of “PL” with the criteria of absolute of log_2_-fold change > 0 and *p* value < 0.05 (Supplementary Table S5). Functions of these genes were related to 119 KEGG pathways (Supplementary Table S6).

### 2.6. Gene Expression Analysis in the Pathway of Biosynthesis of Unsaturated Fatty Acids

**Figure 8:**
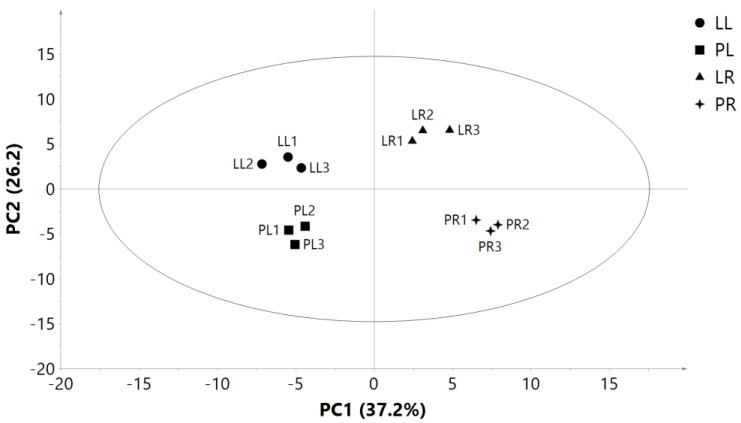
Principal component plot of gene expression participated in the pathway of “biosynthesis of unsaturated fatty acids” from leaves (L) and roots (R) of two purslanes (*Portulaca oleracea*) genotypes (“Pakistan” local (“PL”) and “Liaoning, China” local (“LCL”)). LL: leaves of “LCL”; PL: leaves of “PL”; LR: roots of “LCL”; PR: roots of “LCL”.

**Figure 9:**
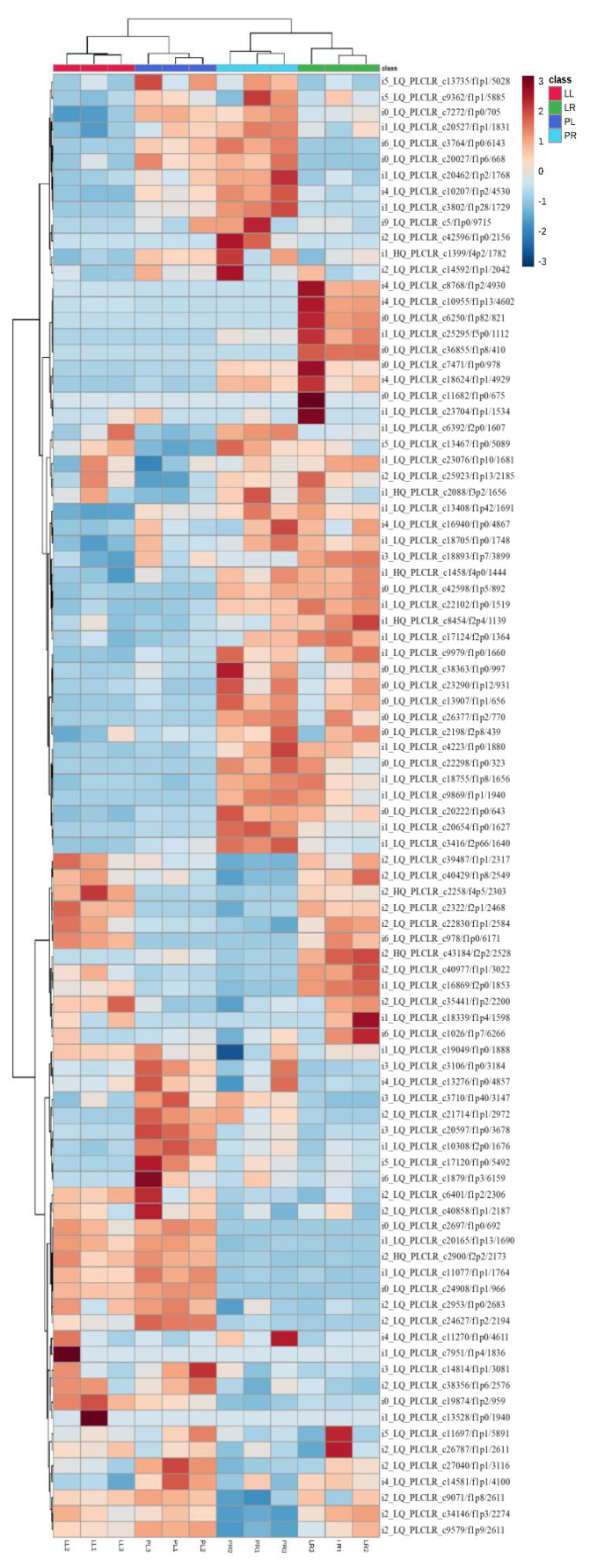
A hierarchically clustered heat map showing the log10 transformed gene expression values in the pathway of “biosynthesis of unsaturated fatty acids” from leaves (L) and roots (R) two purslane (*Portulaca oleracea*) genotypes (“Pakistan” local (“P”) and “Liaoning, China” local (“L”)). Red indicates higher expression, and blue indicates lower expression.

Ninety-four genes were identified to participate in the pathway of “biosynthesis of unsaturated fatty acids” (Table S1). Principle component analysis (PCA) separated these genes expressed in leaves and roots of two purslane genotypes into different principal components. The first and second principal components accounted for 37.25% and 26.2% of the variation, which distinctly separated related gene expressions in leaves and roots of two genotypes (Figure 8). The differentially expressed genes between different organs and genotypes in a heat map (Figure 9) supported our results in PCA analysis. We identified 41 genes with significantly differential expression between “LCL” and “PL” in leaves, of which 30 genes were upregulated and 11 downregulated in “PL” compared with “LCL”. Moreover, 32 genes had a significantly differential expression in roots, of which 13 genes upregulated and 19 downregulated in “PL” compared with “LCL” (Supplementary Table S7).

These 94 genes took parts in acyl-(acyl-carrier-protein) desaturase (K00059, 8 genes), acyl-CoA oxidase (K00232, 24 genes), acyl-coenzyme A thioesterase 1/2/4 (K01068, 5 genes), acyl-(acyl-carrier-protein) desaturase (K03921, 16 genes), acetyl-CoA acyltransferase 1 (K07513, 14 genes), 17 beta-estradiol 17-dehydrogenase/very-long-chain 3-oxoacyl-CoA reductase (K10251, 5 genes), ω-6 fatty acid desaturase/acyl-lipid ω-6 desaturase (K10256, 15 genes), very-long-chain enoyl-CoA reductase (K10258, 6 genes) and very-long-chain (3R)-3-hydroxyacyl-CoA dehydratase (K10703, 1 gene). Among them, genes, which played roles in acyl-(acyl-carrier-protein) desaturase (stearoyl- acyl-carrier-protein desaturase, *SAD*) and ω-6 fatty acid desaturase (*FAD2*), participated in oleic acid (C18:1^Δ9^) and linoleic acid (C18:2^Δ9, 12^) biosynthesis separately, in the ω-3 fatty acid synthesis and shown differential expression in leaves or roots in two genotypes.

16 *SAD* related genes were identified, of which 7 genes had not significantly changed, 6 genes upregulated and 3 downregulated in the leaves, 9 genes had not significantly changed, 5 genes upregulated and 2 downregulated in the roots, of “PL” compared with “LCL”. 4 genes (i1_LQ_PLCLR_c20654/f1p0/1627, i4_LQ_PLCLR_c10207/f1p2/4530, i1_LQ_PLCLR_c3802/f1p28/1729 and i1_LQ_PLCLR_c3416/f2p66/1640) only upregulated in leaves and roots of “PL” and 2 genes (i4_LQ_PLCLR_c10955/f1p13/4602 and i4_LQ_PLCLR_c8768/f1p2/4930) only upregulated in leaves and roots of “LCL”. 14 *FAD2* related genes were identified, of which 10 genes had not significantly changed, 3 genes upregulated and 1 downregulated in the leaves, 11 genes had not significantly changed, 2 genes upregulated and 1 downregulated in the roots, of “PL” compared with “LCL”. 1 *FAD2* (i6_LQ_PLCLR_c3764/f1p0/6143) only upregulated in leaves and roots of “PL” and another FAD2 (i6_LQ_PLCLR_c978/f1p0/6171) only upregulated in leaves and roots of “LCL” (Supplementary Table S7).

## 3. Discussion

Using the PacBio Iso-Seq platform, 15.33 GB subread base and 9,350,222 subreads with 1640, average reads were generated, the total number of transcripts were 132,536, and total genes were 78,559 in purslane. The same with other reports [27,28,29,30], our results have also shown that SMRT technology was an efficient tool for full-length cDNA sequencing and provided a rich resource for further functional genomics analysis in purslane.

All high-quality and unique SMRT transcripts were functionally annotated by seven databases. With GO analysis, transcripts associated through the metabolic process and cellular process in BP, cell and cell part in CC, binding and catalytic activity in MF were enriched in subcategories. Metabolism was also the most important transcript group with KEGG annotation, especially for carbon metabolism, carbon fixation in photosynthetic organisms and pyruvate metabolism. This might provide clues for further study for gene expression and regulation in some specific stress conditions in purslane. LncRNAs are regulated with gene expression, either transcriptional or post-transcriptional levels, during plant growth, development and abiotic and biotic stress [21,31,32,33]. 7289 LncRNA transcripts were identified in purslane with four analytical methods. Their function in purslane needed to be investigated further.

With the gene function interpretation of SMRT transcripts, the second-generation sequencing of purslane was carried out. A large number of differentially expressed genes and related KEGG pathways were found. The biosynthesis of unsaturated fatty acid, especially ω-3 fatty acid, is one kind of nutrient substance and important for the abiotic tolerance of plants. *SAD* and *FAD2* were important genes in the pathway of ω-3 fatty acid biosynthesis. Tissue-specific expressions of *SAD* and *FAD2* were found in some species. Stearoyl-ACP desaturase (SACPD) catalyzes the conversion of stearic acid (18:0) to oleic acid (18:1) during de novo fatty acid biosynthesis [34,35]. Differential expressions of *SAD* were found in seeds, leaves and roots of sacha inchi (*Plukenetia volubilis*) [36]. Eight putative *SAD* isoforms were found in the cacao (*Theobroma cacao*) genome. These eight isoforms displayed diverse expression patterns in various cacao tissues, and these genes exhibited distinct functions in seed and flower development and fatty acid synthesis [37]. Moreover, differential expression of *FAD2* was found in different tissues of sunflower (*Helianthus annuus*) [38], cotton (*Gossypium hirsutum*) [39] and purslane [40]. *SAD* and *FAD2* were highly expressed in leaves and poorly in the roots and the stems of lima bean (*Phaseolus lunatus*) [41]. Our previous results have found that compared with “LCL”, “PL” has a significantly higher content of unsaturated fatty acids [42]. Differential expression of these two genes in leaves and roots of two genotypes of purslane laid the foundation for the further study of gene expression and manipulation in the synthesis of ω-3 fatty acid in purslane.

The composition of fatty acids in plants may also affect plant morphology [43] and the growth rate of a cell population [44]. The *Arabidopsis thaliana* genome had 7 *SAD* isoforms [34]. *SSI2* was a *SAD* isoform, and *SSI2* mutation caused severe growth defects [45]. “PL” has an upright growth habit and “LCL” with a prostrate growth habit. At the same environmental condition, they have different growth rates [46]. It is needed to be verified that if the differential expressions of *SAD* and *FAD2* influenced the growth and development of the purslane genotype.

Abiotic stress regulated the expression of these two genes [41,47,48,49,50,51]. Cold acclimation upregulated *SAD* expression in the cold acclimating species, *Solanum commersonii*, compared with the cold non-acclimating species, *S. tuberosum* [47]. *FAD2* was required for normal growth of *Arabidopsis* at low-temperature [48,49]. High salinity induced *SAD* upregulation in *Hematococcus pluvialis* [50]. Upregulation of *FAD2* was necessary for salt tolerance during seed germination and early seedling growth of *Arabidopsis* under salinity stress [51]. Purslane is a salt-tolerant plant [8]. According to our previous results, “PL” was salt-tolerance and “LCL” was salt-sensitive purslane genotype, respectively [46,52]. Moreover, under 200 mM NaCl stress, ω-3 fatty acid contents in the leaves of “PL” was significantly higher than that in “LCL”. However, the related molecular regulatory mechanism was still unknown. Further studies on elucidating the relationship between *SAD* and *FAD2* expression, ω-3 fatty acid content and salt tolerance will build the foundation for selecting or breeding new genotypes with high ω-3 fatty acid contents and better salt tolerance.

## 4. Materials and Methods

### 4.1. Plants Samples and Treatment

In order to get the full-length transcriptome of purslane and be used for salt-tolerance-related gene function analysis in the future, two purslane genotypes, “PL” and “LCL,” were chosen. After 14 days of sowing, seedlings were transplanted into plastic hydroponic boxes in a greenhouse on 23 March 2018 at the School of Agriculture and Biology, Shanghai Jiao Tong University, China.

Hydroponic system (absenteeism of O_2_) was chosen from our previous experiments [46]. For decreasing the environmental influence, 12 plants of selected genotypes remained in the same box as per replicate. The experiment was repeated three times. 15 L quarter-strength of Hoagland’s solution with an electrical conductivity of 4.0 dS m^−1^ and a pH of 5.8 were put in each plastic box, and quarter-strength of Hoagland’s solutions was replaced 2 times per week. The plantlets were fully mature in a greenhouse with a day temperature of 28 ± 2 °C and night temperature of 16 ± 2 °C, 70–80% relative humidity and 400 μmol·m^−2^·s^−1^ photosynthetically active radiation. Plant tissue was collected from biological replicates in each box after two weeks of transplant. The composed tissues were directly ice-covered by liquid nitrogen, reserved at –80 °C until RNA isolation.

### 4.2. RNA Extraction, Library Construction and SMART Sequencing

Complete RNA samples (0.2 g leaves or roots from “PL” and “LCL” were mixed together) were extracted via RNeasy Plus mini kit (Qiagen, Valencia, CA, USA). After monitoring the deprivation and contamination percentage using agarose gel, RNA integrity (OD260/280) was checked with Nanodrop ND-1000 spectrophotometer (Nanodrop Technologies, Rockland, DE, USA). RNA level and RNA authenticity were restrained distinctly by Qubit^®^ RNA Assay Kit and RNA Nano 6000 Assay Kit(Thermo Fisher Scientific, Waltham, MA, USA).

Disinfected RNA was secluded from total RNA using oligo (dT) enriches mRNA containing poly-(A) beads. The SMART PCR cDNA synthesis kit (Clontech, CA, USA) was used for cDNA synthesis. For the selection of differently sized full-length cDNA and for the construction of cDNA libraries, the BluePippin^®^ (SageScience, Beverly, MA, USA) was used. After screened by Blue Pippin, the fragments were subjected to large-scale PCR to obtain sufficient total cDNA by using a Qubit fluorometer (Life Technologies, Carlsbad, CA, USA). Library uniqueness was kept constant by using Agilent Bioanalyzer 2100 system. SMRT sequencing was achieved via PacBio’s real-time sequencer using C2 sequencing reagents. Quality filtering and error correction followed the methods of the literature [53].

### 4.3. Functional Annotation of Transcripts

We identified functional annotations matching each unique transcript searching NR [54], NT, Pfam (http://pfam.xfam.org, accessed on 20 January 2021), KOG (http://www.ncbi.nlm.nih.gov/COG/, accessed on 20 January 2021) [55], Swiss-Prot (http://www.ebi.ac.uk/uniprot/, accessed on 20 January 2021) [56], KEGG (http://www.genome.jp/kegg/, accessed on 20 January 2021) [57] and GO [58]. We used the software of BLAST and NT data storage analysis, software of Diamond BLASTX v2.7.1 and set e-value “1e-10” in KEGG, Swiss-Prot, KOG, NR. and the software of Hmmscan in Pfam database analysis.

### 4.4. Identification of Transcript Factors, Long Non-Coding RNAs and Simple Sequence Repeat

Plant transcription factors were predicted using iTAK v1.7a (https://github.com/kentnf/iTAK/, accessed on 20 January 2021) [26]. Four tools, CNCIv2 (https://github.com/www-bioinfo-org/CNCI, accessed on 20 January 2021) [59], CPCvcpc-0.9-r2 (http://cpc.cbi.pku.edu.cn/, accessed on 20 January 2021) with e-value ‘1e-10′ [60], Pfam-scan (E 0.001-domE 0.001) [61] and PLEKv1.2 (https://sourceforge.net/projects/plek/, accessed on 20 January 2021) with min length 200 [62] were chosen to predict candidate LncRNAs. Transcripts forecasted without coding potential were candidate set of LncRNAs. SSRs were identified by MISA v1.0 (http://pgrc.ipk-gatersleben.de/misa/, accessed on 20 January 2021) [63] with default parameters. MISA can identify seven kinds of SSR types.

### 4.5. Illumina cDNA Library Construction and Second-Generation Sequencing Analysis

Total RNA was extracted from leaves or roots with three independent biological replicates obtained from each genotype. Next, RNA purity was checked using the NanoPhotometer^®^ spectrophotometer (IMPLEN, Westlake Village, CA, USA), and RNA integrity was assessed using the RNA Nano 6000 Assay Kit of the Agilent Bioanalyzer 2100 system (Agilent Technologies, Santa Clara, CA, USA). A total amount of 1.5 μg RNA per sample was used as input material for the RNA sample preparations. Sequencing libraries were generated using NEBNext^®^ Ultra™ RNA Library Prep Kit for Illumina^®^ (NEB, Ipswich, MA, USA) following the manufacturer’s recommendations, and index codes were added to attribute sequences to each sample. The clustering of the index-coded samples was performed on a cBot cluster generation system using TruSeq PE Cluster Kit v3-cBot-HS (Illumia, San Diego, CA, USA) according to the manufacturer’s instructions. After cluster generation, the library preparations were sequenced on an Illumina HiSeq XTEN platform (San Diego, CA, USA), and paired-end reads were generated.

CD-HIT software was used to remove redundant and similar sequences [64], a nonredundant transcriptome generated by SMRT sequencing was used as a REF, then clean reads of each sample obtained from Illumina sequencing were compared to the REF. RSEM software was used in this process [65]. FPKM conversion was performed to analyze the gene expression level. Differential expression analyses of genes in leaves or roots between “PL” and “LCL” were performed using the DESeq R package (1.10.1). The resulting *p* values were adjusted using Benjamini–Hochberg’s approach [66] for controlling the false discovery rate. DEGs were selected with the criteria of absolute of log_2_-fold change > 0 and *p*-value < 0.05 by comparing differences between two genotypes of leaves or roots. GO enrichment analysis and KEGG pathway enrichment analysis were implemented to test the statistical enrichment of DEGs in KEGG pathways, respectively.

### 4.6. Quantitative RT–PCR Analysis

qRT–PCR was carried out on a Mastercycler ep realplex, real-time PCR system (Eppendorf, Hamburg, Germany) using Bestar SYBR Green qPCR Mastermix (DBI, Bioscience Inc., Hamburg, Germany) [67]. Reactions were performed at 94 °C for 40 s, 30 cycles of 94 °C for 10 s, 54 °C for 30 s and 72 °C for 90 s. Three biological replicates were applied. The specific primers were designed using the Primer Premier 5.0 software. The primer sequences are listed in Supplementary Table S8. Actin was used as the reference genes. The target genes’ relative expression levels were calculated using the 2^−ΔΔCT^ approach [68].

## 5. Conclusions

In this scenario, we finished RNA-seq in purslane with SMRT technology. Related results provided significant information for enlightening the whole-genome annotation and transcriptomic characterization that might be useful for genetic manipulation of purslane genotypes under abiotic stresses. With the function of gene function interpretation of RNA-seq technology, we finished Illumina sequencing of leaves and roots of two purslane genotypes and found differential expression of some genes, especially *FAD2* and *SAD*, may be related with ω-fatty acid contents of special genotypes of purslane.

## Figures and Tables

**Table 1:** Statistics of annotation results for unigenes of two purslanes (*Portulaca oleracea*) genotypes.

Sample	Raw Reads	Clean Reads	Mapping Rate (%)
**Leaves**			
“LCL”, 1	62,777,144	47,789,582	76.13
“LCL”, 2	60,625,062	45,707,454	75.39
“LCL”, 3	54,712,526	40,774,382	74.52
“PL”, 1	54,782,840	41,921,110	76.52
“PL”, 2	61,500,192	47,256,666	76.84
“PL”, 3	59,127,304	44,841,760	75.84
**Roots**			
“LCL”, 1	58,010,278	34,114,332	58.81%
“LCL”, 2	56,153,952	34,729,076	61.85%
“LCL”, 3	59,146,128	37,448,562	63.32%
“PL”, 1	63,212,838	38,289,822	60.57%
“PL”, 2	48,132,318	28,489,398	59.19%
“PL”, 3	42,508,608	24,481,842	57.59%

## Data Availability

Raw CCS reads are available at the SRA database of NCBI GenBank under the accession PRJNA522036.

## Supplementary Materials

The following are available online at https://www.mdpi.com/article/10.3390/plants10040655/s1, Table S1: Function annotation of all corrected isoforms in seven databases by SMRT. Table S2: Transcription factor prediction of Iso-Seq reads. Table S3: LncRNAs and mRNAs from Iso-Seq. Table S4: SSR and corresponding primer. Table S5: Differentially expressed transcripts in four comparisons (Leaves or root, “LCL” vs. “PL”, up or down). Table S6: KEGG pathway enrichment results of upregulated and downregulated pathways in leaves and roots of two purslane genotypes (“LCL” and “PL”). Table S7: Gene expressions of participated in the biosynthesis of unsaturated fatty acids in leaves and roots of two genotypes (“LCL” and “PL”) of purslane (*Portulaca oleracea*). Table S8: Primers used for qRT–PCR analysis of differential genes in purslane (*Portulaca oleracea*).
