# Supplementary material for: Single-Molecule Long-Read Sequencing of Purslane (Portulaca oleracea) and Differential Gene Expression Related with Biosynthesis of Unsaturated Fatty Acids
Source: Plants (Basel). 2021 Mar 30;10(4):655. doi: 10.3390/plants10040655 (PMC8066459; doi:10.3390/plants10040655)
Supplement: Supplementary file 1 [file plants-10-00655-s001.zip › Supplementary materials/Supplementary table 7.doc]

**Table S7** Gene expressions of participated in biosynthesis of unsaturated fatty acids in leaves and roots of two genotypes (‘LCL’ and ‘PL’) of purslane (*Portulaca oleracea*)

| **No.** | **Transcript ID** | **Gene name** | **Function description (Species)** | **FPKM** | | | |
| --- | --- | --- | --- | --- | --- | --- | --- |
|  |  |  |  | **Leaves** | | **Roots** | |
|  |  |  |  | **‘LCL’** | **‘PL’** | **‘LCL’** | **‘PL’** |
| **K00059 [**3-oxoacyl-(acyl-carrier protein) reductase**]** | | | | | | | |
| 1 | i0_LQ_PLCLR_c23290/f1p12/931 |  | Short-chain type dehydrogenase/reductase (*Picea abies*) | 5.21 ± 0.60 | 5.33 ± 1.38 | 15.23 ± 3.62 | 21.38 ± 4.67 |
| 2 | i0_LQ_PLCLR_c38363/f1p0/997 |  |  | 7.07 ± 0.36 | 10.56 ± 2.22 | 17.58 ± 4.06 | 31.53 ± 7.51 |
| 3 | i1_LQ_PLCLR_c25295/f5p0/1112 |  |  | 0.00 ± 0.00 | 0.00 ± 0.00 | 287.89 ± 47.80 *^1^ | 85.64 ± 80.7 |
| 4 | i0_LQ_PLCLR_c36855/f1p8/410 |  |  | 0.11 ± 0.11 | 0.00 ± 0.00 | 246.42 ± 8.35 * | 0.00 ± 0.00 |
| 5 | i0_LQ_PLCLR_c6250/f1p82/821 |  |  | 0.05 ± 0.05 | 0.00 ± 0.00 | 243.25 ± 43.48 * | 0.00 ± 0.00 b |
| 6 | i0_LQ_PLCLR_c7471/f1p0/978 |  |  | 0.79 ± 0.13 | 2.20 ± 0.35 * | 29.22 ± 11.99 | 17.80 ± 1.02 |
| 7 | i1_LQ_PLCLR_c4223/f1p0/1880 | *CLKR27* | 3-oxoacyl-[acyl-carrier-protein] reductase, chloroplastic (*Cuphea lanceolate*) | 22.17 ± 0.36 | 23.25 ± 0.71 | 34.99 ± 1.48 | 38.44 ± 4.32 |
| 8 | i1_LQ_PLCLR_c17124/f2p0/1364 |  |  | 34.97 ± 3.01 | 35.24 ± 1.78 | 60.51 ± 2.72 | 48.94 ± 4.98 |
| **K00232 (**acyl-CoA oxidase**)** | | | | | | | |
| 9 | i0_LQ_PLCLR_c13216/f1p16/990 | *ACX1* | Peroxisomal acyl-coenzyme A oxidase 1 (*Arabidopsis thaliana*) | 0.00 ± 0.00 | 0.00 ± 0.00 | 0.00 ± 0.00 | 0.00 ± 0.00 |
| 10 | i1_LQ_PLCLR_c7951/f1p4/1836 |  |  | 0.01 ± 0.01 | 0.00 ± 0.00 | 0.00 ± 0.00 | 0.00 ± 0.00 |
| 11 | i2_LQ_PLCLR_c6401/f1p2/2306 |  |  | 4.69 ± 0.26 | 4.89 ± 1.63 | 1.49 ± 0.53 | 1.66 ± 0.11 |
| 12 | i2_LQ_PLCLR_c38356/f1p6/2576 |  |  | 0.27 ± 0.07 | 0.29 ± 0.05 | 0.11 ± 0.02 | 0.12 ± 0.04 |
| 13 | i2_LQ_PLCLR_c40858/f1p1/2187 |  |  | 0.19 ± 0.04 | 0.28 ± 0.07 | 0.14 ± 0.04 | 0.09 ± 0.03 |
| 14 | i2_LQ_PLCLR_c40429/f1p8/2549 |  |  | 2.37 ± 0.35 | 1.46 ± 0.20 | 2.60 ± 0.40 * | 0.35 ± 0.13 |
| 15 | i2_LQ_PLCLR_c9071/f1p8/2611 |  |  | 35.12 ± 0.42 | 36.98 ± 0.51 * | 33.84 ± 2.18 | 27.25 ± 1.30 |
| 16 | i2_LQ_PLCLR_c9579/f1p9/2611 |  |  | 36.37 ± 0.59 | 41.50 ± 0.35 * | 36.41 ± 2.12 * | 24.84 ± 0.75 |
| 17 | i2_HQ_PLCLR_c2258/f4p5/2303 |  |  | 21.65 ± 3.47 * | 3.29 ± 0.35 b | 12.47 ± 1.03* | 1.88 ± 0.52 |
| 18 | i1_LQ_PLCLR_c9869/f1p1/1940 | *ACX* | Acyl-coenzyme A oxidase, peroxisomal (*Cucurbita maxima*) | 0.00 ± 0.00 | 0.00 ± 0.00 | 8.54 ± 2.40 | 13.37 ± 1.34 |
| 19 | i2_LQ_PLCLR_c39487/f1p1/2317 | *ACX2* | Acyl-coenzyme A oxidase 2, peroxisomal (*Arabidopsis thaliana*) | 4.19 ± 0.64 | 2.69 ± 0.20 | 3.72 ± 0.44 * | 1.22 ± 0.07 |
| 20 | i2_LQ_PLCLR_c22830/f1p1/2584 |  |  | 4.42 ± 0.82 * | 2.01 ± 0.06 b | 4.37 ±0.60 * | 0.94 ± 0.30 |
| 21 | i2_LQ_PLCLR_c2322/f2p1/2468 |  |  | 13.38 ± 1.24 * | 5.10 ± 0.11 b | 11.54 ±0.58 * | 4.47 ± 0.88 |
| 22 | i2_LQ_PLCLR_c42596/f1p0/2156 | *ACX3* | Acyl-coenzyme A oxidase 3, peroxisomal (*Arabidopsis thaliana*) | 0.00 ± 0.00 | 0.00 ± 0.00 | 0.00 ± 0.00 | 0.19 ± 0.08 |
| 23 | i4_LQ_PLCLR_c16940/f1p0/4867 |  |  | 0.01 ± 0.01 | 0.09 ± 0.04 | 0.15 ± 0.04 | 0.16 ± 0.08 |
| 24 | i4_LQ_PLCLR_c14581/f1p1/4100 |  |  | 0.41 ± 0.09 | 1.17 ± 0.16 * | 0.90 ± 0.05 | 0.56 ± 0.21 |
| 25 | i2_HQ_PLCLR_c43184/f2p2/2528 |  |  | 19.22 ± 0.19 | 24.09 ± 1.01 * | 42.89 ± 3.65 * | 15.99 ± 0.49 |
| 26 | i1_LQ_PLCLR_c20462/f1p2/1768 | *ACX3.2* | Putative acyl-coenzyme A oxidase 3.2, peroxisomal (*Arabidopsis thaliana*) | 0.09 ± 0.02 | 0.26 ± 0.05 * | 0.11 ±0.04 | 0.50 ± 0.08 * |
| 27 | i3_LQ_PLCLR_c14814/f1p1/3081 | *ACX4* | Acyl-coenzyme A oxidase 4, peroxisomal (*Arabidopsis thaliana*) | 0.06 ± 0.03 | 0.10 ± 0.03 | 0.02 ± 0.00 | 0.03 ± 0.02 |
| 28 | i1_LQ_PLCLR_c20527/f1p1/1831 |  |  | 17.91 ± 0.98 | 24.50 ± 1.39 * | 22.41 ± 1.39 | 28.63 ± 0.95 * |
| 29 | i2_LQ_PLCLR_c27040/f1p1/3116 |  |  | 2.51 ± 0.35 | 5.13 ± 0.46 * | 3.05 ± 0.55 | 1.65 ± 0.19 |
| 30 | i2_LQ_PLCLR_c40977/f1p1/3022 |  |  | 1.95 ± 0.50 * | 0.54 ± 0.08 b | 3.54 ±0.37 * | 0.18 ± 0.09 |
| 31 | i1_LQ_PLCLR_c16869/f2p0/1853 |  |  | 23.53 ± 1.71 * | 13.20 ± 0.97 b | 39.84 ±1.97 * | 7.09 ± 0.58 |
| 32 | i0_LQ_PLCLR_c7272/f1p0/705 | *At3g06690* | Putative acyl-coenzyme A oxidase At3g06690 (*Arabidopsis thaliana*) | 7.50 ± 1.16 | 18.08 ± 0.59 * | 13.34 ± 0.51 | 18.37 ± 1.42 * |
| **K01068 (**acyl-coenzyme A thioesterase 1/2/4**)** | | | | | | | |
| 33 | i2_LQ_PLCLR_c2953/f1p0/2683 |  | NA | 0.61 ± 0.10 | 0.74 ± 0.04 | 0.36 ± 0.03 | 0.34 ± 0.10 |
| 34 | i6_LQ_PLCLR_c1026/f1p7/6266 |  | NA | 0.51 ± 0.06 | 0.50 ± 0.01 | 0.67 ± 0.12 | 0.48 ± 0.06 |
| 35 | i2_LQ_PLCLR_c26787/f1p1/2611 |  | NA | 0.14 ± 0.01 | 0.12 ± 0.02 | 0.12 ± 0.09 | 0.07 ± 0.02 |
| 36 | i1_LQ_PLCLR_c19049/f1p0/1888 |  | NA | 3.23 ± 0.03 | 3.17 ± 0.26 | 2.83 ± 0.12 | 2.35 ± 0.62 |
| 37 | i5_LQ_PLCLR_c9362/f1p1/5885 |  | NA | 0.57 ± 0.06 | 1.04 ± 0.08 * | 0.91 ±0.14 | 1.23 ± 0.40 |
| **K03921 [**acyl-(acyl-carrier-protein) desaturase**]** | | | | | | | |
| 38 | i4_LQ_PLCLR_c11270/f1p0/4611 | *SAD* | Stearoyl-[acyl-carrier-protein] 9-desaturase, chloroplastic (*Simmondsia chinensis*) | 0.01 ± 0.01 | 0.01 ± 0.00 | 0.01 ± 0.00 | 0.02 ± 0.01 |
| 39 | i9_LQ_PLCLR_c5/f1p0/9715 |  |  | 0.00 ± 0.00 | 0.01 ± 0.01 | 0.01 ± 0.00 | 0.02 ± 0.01 |
| 40 | i1_HQ_PLCLR_c2088/f3p2/1656 |  |  | 32.10 ± 4.34 | 23.85 ± 1.63 | 33.28 ± 4.82 | 38.26 ± 4.53 |
| 41 | i1_LQ_PLCLR_c20654/f1p0/1627 |  |  | 0.76 ± 0.27 | 4.11 ± 0.42 * | 18.80 ± 2.90 | 61.81 ± 4.43 * |
| 42 | i4_LQ_PLCLR_c10207/f1p2/4530 |  |  | 0.21 ± 0.04 | 1.22 ± 0.08 * | 0.67 ±0.10 | 2.11 ± 0.18 * |
| 43 | i1_LQ_PLCLR_c3802/f1p28/1729 |  |  | 7.73 ± 0.48 | 41.73 ± 1.67 * | 20.01 ± 0.94 | 97.62 ± 8.37 * |
| 44 | i1_LQ_PLCLR_c3416/f2p66/1640 |  |  | 10.73 ± 0.45 | 19.11 ± 1.40 * | 20.40 ± 1.80 | 43.58 ± 1.91 * |
| 45 | i4_LQ_PLCLR_c10955/f1p13/4602 |  |  | 0.53 ± 0.09 * | 0.00 ± 0.00 b | 13.56 ±2.92 * | 0.09 ± 0.01 |
| 46 | i4_LQ_PLCLR_c8768/f1p2/4930 |  |  | 0.04 ± 0.01 * | 0.00 ± 0.00 b | 1.35 ±0.38 * | 0.00 ± 0.00 |
| 47 | i4_LQ_PLCLR_c18624/f1p1/4929 |  | Stearoyl-[acyl-carrier-protein] 9-desaturase, chloroplastic (*Solanum commersonii*) | 0.00 ± 0.00 | 0.04 ± 0.02 | 0.52 ± 0.16 | 0.43 ± 0.03 |
| 48 | i5_LQ_PLCLR_c13467/f1p0/5089 |  |  | 3.88 ± 0.42 * | 1.67 ± 0.11 b | 3.31 ±0.38 | 4.62 ± 0.51 |
| 49 | i5_LQ_PLCLR_c13735/f1p1/5028 |  |  | 0.03 ± 0.02 | 0.18 ± 0.06 | 0.02 ± 0.02 | 0.14 ± 0.04 * |
| 50 | i1_LQ_PLCLR_c20165/f1p13/1690 |  | Stearoyl-[acyl-carrier-protein] 9-desaturase, chloroplastic (*Spinacia oleracea*) | 11.09 ± 0.97 | 12.02 ± 0.54 | 3.10 ± 0.05 | 2.83 ± 0.36 |
| 51 | i2_HQ_PLCLR_c2900/f2p2/2173 |  |  | 32.45 ± 3.39 | 34.91 ± 1.77 | 9.02 ± 0.40 | 9.34 ± 0.75 |
| 52 | i2_LQ_PLCLR_c24627/f1p2/2194 |  |  | 3.77 ± 0.43 | 8.28 ± 0.24 * | 1.07 ±0.04 | 1.43 ± 0.17 |
| 53 | i1_LQ_PLCLR_c11077/f1p1/1764 |  | Stearoyl-[acyl-carrier-protein] 9-desaturase, chloroplastic (*Helianthus annuus*) | 10.64 ± 0.76 | 14.12 ± 0.78 * | 3.46 ±0.30 | 3.34 ± 0.22 |
| **K07513 (**acetyl-CoA acyltransferase 1**)** | | | | | | | |
| 54 | i2_LQ_PLCLR_c14592/f1p1/2042 | *PED1* | 3-ketoacyl-CoA thiolase 2, peroxisomal (*Arabidopsis thaliana*) | 0.08 ± 0.03 | 0.21 ± 0.04 | 0.16 ± 0.06 | 0.26 ± 0.12 |
| 55 | i4_LQ_PLCLR_c13276/f1p0/4857 |  |  | 0.56 ± 0.03 | 0.79 ± 0.11 | 0.43 ± 0.01 | 0.57 ± 0.21 |
| 56 | i0_LQ_PLCLR_c22298/f1p0/323 |  |  | 0.00 ± 0.00 | 0.00 ± 0.00 | 50.94 ± 19.52 | 89.15 ± 11.51 |
| 57 | i3_LQ_PLCLR_c20597/f1p0/3678 |  |  | 1.04 ± 0.02 | 2.93 ± 0.24 * | 0.96 ±0.09 | 1.36 ± 0.18 |
| 58 | i1_LQ_PLCLR_c13408/f1p42/1691 |  |  | 43.65 ± 1.44 | 79.85 ± 2.22 * | 93.99 ± 5.85 | 98.75 ± 8.44 |
| 59 | i3_LQ_PLCLR_c3106/f1p0/3184 |  |  | 1.51 ± 0.09 | 3.52 ± 0.50 * | 0.91 ± 0.10 | 2.07 ± 1.00 |
| 60 | i3_LQ_PLCLR_c3710/f1p40/3147 |  |  | 0.03 ± 0.02 | 0.13 ± 0.03 * | 0.01 ± 0.01 | 0.10 ± 0.01 * |
| 61 | i2_LQ_PLCLR_c21714/f1p1/2972 |  |  | 0.01 ± 0.01 | 0.21 ± 0.02 * | 0.01 ± 0.01 | 0.12 ± 0.03 * |
| 62 | i1_LQ_PLCLR_c10308/f2p0/1676 |  |  | 33.20 ± 0.69 | 49.79 ± 1.15 * | 34.87 ± 1.39 | 37.72 ± 1.37 |
| 63 | i0_LQ_PLCLR_c20027/f1p6/668 |  |  | 0.14 ± 0.14 | 0.93 ± 0.17 * | 0.00 ±0.00 | 1.09 ± 0.15 * |
| 64 | i5_LQ_PLCLR_c17120/f1p0/5492 |  |  | 0.00 ± 0.00 | 0.31 ± 0.08 * | 0.04 ±0.02 | 0.08 ± 0.03 |
| 65 | i2_LQ_PLCLR_c35441/f1p2/2200 |  |  | 3.78 ± 0.53 * | 1.56 ± 0.37 b | 3.27 ±0.73 | 1.43 ± 0.51 |
| 66 | i2_LQ_PLCLR_c34146/f1p3/2274 | *KAT1* | 3-ketoacyl-CoA thiolase 1, peroxisomal (*Arabidopsis thaliana*) | 35.40 ± 2.69 | 35.28 ± 1.35 | 38.06 ± 2.72 * | 16.71 ± 0.58 |
| 67 | i1_LQ_PLCLR_c18705/f1p0/1748 | *KAT5* | 3-ketoacyl-CoA thiolase 5, peroxisomal (*Arabidopsis thaliana*) | 2.40 ± 0.31 | 4.87 ± 0.81 * | 6.17 ± 0.36 | 6.42 ± 0.99 |
| **K10251 (**17 beta-estradiol 17-dehydrogenase / very-long-chain 3-oxoacyl-CoA reductase**)** | | | | | | | |
| 68 | i0_LQ_PLCLR_c26377/f1p2/770 | *KCR1* | Very-long-chain 3-oxoacyl-CoA reductase 1 (*Arabidopsis thaliana*) | 0.09 ± 0.05 | 0.18 ± 0.05 | 2.23 ± 0.95 | 3.76 ± 0.22 |
| 69 | i0_LQ_PLCLR_c11682/f1p0/675 |  |  | 0.00 ± 0.00 | 0.00 ± 0.00 | 0.08 ± 0.08 | 0.00 ± 0.00 |
| 70 | i0_LQ_PLCLR_c42598/f1p5/892 |  |  | 4.47 ± 1.36 | 3.78 ± 0.45 | 36.48 ± 2.37 | 32.39 ± 1.99 |
| 71 | i1_LQ_PLCLR_c22102/f1p0/1519 |  |  | 31.16 ± 3.39 | 32.39 ± 3.13 | 82.67 ± 4.04 * | 66.98 ± 0.74 |
| 72 | i0_LQ_PLCLR_c20222/f1p0/643 |  |  | 3.50 ± 0.54 | 6.86 ± 0.23 * | 15.53 ± 1.55 | 21.35 ± 2.30 |
| **K10256 [**omega-6 fatty acid desaturase / acyl-lipid omega-6 desaturase (Delta-12 desaturase)**]** | | | | | | | |
| 73 | i5_LQ_PLCLR_c11697/f1p1/5891 | *FADX* | Delta (12) acyl-lipid conjugase (11E, 13E-forming) (*Momordica charantia*) | 0.04 ± 0.00 | 0.06 ± 0.02 | 0.05 ± 0.04 | 0.03 ± 0.01 |
| 74 | i2_LQ_PLCLR_c25923/f1p13/2185 | *FAD2* | Delta (12)-fatty-acid desaturase FAD2 (*Vernicia fordii*) | 126.34 ± 17.37 | 83.35 ± 9.17 | 139.65 ± 15.00 | 128.33 ± 2.98 |
| 75 | i1_LQ_PLCLR_c23076/f1p10/1681 |  |  | 23.71 ± 3.97 | 17.91 ± 3.13 | 27.40 ± 1.60 | 24.01 ± 1.30 |
| 76 | i1_LQ_PLCLR_c18755/f1p8/1656 |  |  | 17.79 ± 1.34 | 17.30 ± 1.07 | 36.57 ± 5.35 | 42.71 ± 1.48 |
| 77 | i0_LQ_PLCLR_c13907/f1p1/656 |  |  | 6.30 ± 0.89 | 14.43 ± 2.92 | 49.18 ± 12.88 | 77.53 ± 9.18 |
| 78 | i1_LQ_PLCLR_c23704/f1p1/1534 |  |  | 0.01 ± 0.01 | 0.02 ± 0.02 | 0.07 ± 0.07 | 0.01 ± 0.01 |
| 79 | i1_LQ_PLCLR_c20236/f1p0/1629 |  |  | 0.00 ± 0.00 | 0.00 ± 0.00 | 0.00 ± 0.00 | 0.00 ± 0.00 |
| 80 | i1_LQ_PLCLR_c18339/f1p4/1598 |  |  | 0.02 ± 0.01 | 0.00 ± 0.00 | 0.04 ± 0.03 | 0.00 ± 0.00 |
| 81 | i1_LQ_PLCLR_c13528/f1p0/1940 |  |  | 0.01 ± 0.01 | 0.00 ± 0.00 | 0.00 ± 0.00 | 0.00 ± 0.00 |
| 82 | i6_LQ_PLCLR_c1879/f1p3/6159 |  |  | 0.00 ± 0.00 | 0.03 ± 0.01 | 0.00 ± 0.00 | 0.01 ± 0.01 |
| 83 | i1_HQ_PLCLR_c1399/f4p2/1782 |  |  | 60.52 ± 5.37 | 120.54 ± 3.31 * | 83.13 ±15.83 | 137.14 ± 32.47 |
| 84 | i1_LQ_PLCLR_c9979/f1p0/1660 |  |  | 4.31 ± 0.74 | 14.81 ± 1.50 * | 40.35 ±10.89 | 42.74 ± 8.40 |
| 85 | i6_LQ_PLCLR_c3764/f1p0/6143 |  |  | 0.16 ± 0.03 | 2.76 ± 0.25 * | 0.09 ±0.04 | 4.24 ± 0.32 * |
| 86 | i6_LQ_PLCLR_c978/f1p0/6171 |  |  | 0.62 ± 0.05 * | 0.00 ± 0.00 b | 0.52 ±0.11 * | 0.00 ± 0.00 |
| 87 | i1_LQ_PLCLR_c6392/f2p0/1607 |  |  | 32.81 ± 12.16 | 13.80 ± 1.45 | 25.57± 1.13 | 49.14 ± 2.23 * |
| **K10258 (**very-long-chain enoyl-CoA reductase**)** | | | | | | | |
| 88 | i0_LQ_PLCLR_c2697/f1p0/692 |  | NA | 29.19 ± 6.22 | 37.21 ± 2.26 | 0.00 ± 0.00 | 0.00 ± 0.00 |
| 89 | i0_LQ_PLCLR_c2198/f2p8/439 | *ECR* | Very-long-chain enoyl-CoA reductase (*Arabidopsis thaliana*) | 14.98 ± 4.03 | 17.38 ± 0.76 | 23.75 ± 5.73 | 28.51 ± 3.38 |
| 90 | i1_HQ_PLCLR_c1458/f4p0/1444 |  |  | 40.29 ± 2.64 | 48.80 ± 2.45 | 65.68 ± 2.10 | 62.14 ± 4.17 |
| 91 | i3_LQ_PLCLR_c18893/f1p7/3899 |  |  | 5.09 ± 0.39 | 6.73 ± 0.51 | 8.26 ± 0.17 * | 6.29 ± 0.02 |
| 92 | i0_LQ_PLCLR_c19874/f1p2/959 | *art-1* | Probable very-long-chain enoyl-CoA reductase art-1 (*Caenorhabditis elegans*) | 16.66 ± 1.86 * | 10.12 ± 0.71 b | 5.16 ±0.17 * | 2.29 ± 0.46 |
| 93 | i0_LQ_PLCLR_c24908/f1p1/966 | *DET2* | Steroid 5-alpha-reductase DET2 (*Gossypium hirsutum*) | 54.87 ± 2.10 | 70.20 ± 1.31 * | 3.30 ±0.53 | 4.65 ± 0.79 |
| **K10703 (**very-long-chain (3R)-3-hydroxyacyl-CoA dehydratase**)** | | | | | | | |
| 94 | i1_HQ_PLCLR_c8454/f2p4/1139 | *PAS2A* | Very-long-chain (3R)-3-hydroxyacyl- CoA dehydratase PASTICCINO 2A (*Oryza sativa* subsp. *Japonica*) | 36.35 ± 5.30 | 32.22 ± 2.06 | 66.33 ± 6.37 | 45.66 ± 5.29 |

^1^ The asterisk represents statistical significance for comparison between gene expression of two genotypes in leaves or roots (*P* = 0.05).
