# Supplementary material for: Single-Molecule Long-Read Sequencing of Purslane (Portulaca oleracea) and Differential Gene Expression Related with Biosynthesis of Unsaturated Fatty Acids
Source: Plants (Basel). 2021 Mar 30;10(4):655. doi: 10.3390/plants10040655 (PMC8066459; doi:10.3390/plants10040655)
Supplement: Supplementary file 1 [file plants-10-00655-s001.zip › Supplementary materials/Supplementary table 8.doc]

**Table S8** Primers used for qRT-PCR analysis of differential genes in purslane (*Portulaca oleracea*)

| Gene ID | Gene name | KEGG pathway | Function Description | Primers | |
| --- | --- | --- | --- | --- | --- |
| i2_LQ_PLCLR_c14761/f1p2/2158 | *FPGS2* | Folate biosynthesis | Folate biosynthesis | F | CAACTGCATGGATGTGAGAA |
|  |  |  |  | R | CAGGACAAGTTGGTTGTAGG |
| i4_LQ_PLCLR_c18937/f1p3/5081 | *PAO4* | Arginine and proline metabolism | beta-Alanine metabolism | F | AGTTGATTCTTGCATCGCTT |
|  |  |  |  | R | CCAAGTCTGTCACGAGATTC |
| i1_LQ_PLCLR_c10511/f1p5/1691 | *ADH1* | α-linolenic acid metabolism | Alcohol dehydrogenase class-P | F | CGACTTACCTTCTGTTGTGG |
|  |  |  |  | R | CAAAAACCAAGTCCTTGCAC |
| i1_HQ_PLCLR_c25814/f23p0/1497 | *ADH1* | α-linolenic acid metabolism | Alcohol dehydrogenase class-P | F | AGCATTTGATTTCTGCGAGT |
|  |  |  |  | R | ACGGACTTCGTTTTTCTGAG |
| i2_LQ_PLCLR_c41265/f1p4/2925 | *ADH1* | α-linolenic acid metabolism | Alcohol dehydrogenase class-P | F | GGCACACAAATCCCAGTTAT |
|  |  |  |  | R | CTCAGCTAGCACACCAATTT |
| i3_LQ_PLCLR_c16229/f2p0/3016 | *ETR2* | Plant hormone signal transduction | Ethylene receptor 2 | F | CCTTCCTTTTGTGACTCAGG |
|  |  |  |  | R | TTACATCTAGGGAACCCACC |
|  | *Actin* |  |  | F | CCTCTCTCGGTGAGGA |
|  |  |  |  | R | TCCAGGCTGTCCTGTC |
